# Giant and multiple jejunal diverticula presenting as peritonitis a significant challenging disorder

**Published:** 2012-09-25

**Authors:** R Singal, S Gupta, A Airon

**Affiliations:** * Department of Surgery, Maharishi Markandeshwer Institute of Medical Sciences and Research, Mullana, Haryana, India; ** Department of Radiodiagnosis and Imaging, Maharishi Markandeshwer Institute of Medical Sciences and Research, Mullana, Haryana, India; *** Department of Medicine, Maharishi Markandeshwer Institute of Medical Sciences and Research, Mullana, Haryana, India

**Keywords:** acute abdomen, ileum, small bowel diverticulum, perforation, surgery

## Abstract

Jejunal diverticula are an uncommon acquired disease that is usually silent and asymptomatic. When symptomatic, they present with chronic nonspecific symptoms like pain, nausea, malnutrition and sometimes with acute presentation like gastrointestinal hemorrhage, peritonitis and obstruction. The majority of complications seen as an acute abdomen similar to appendicitis, cholecystitis or colonic diverticulitis but they also may appear with atypical symptoms. We are presenting a 63-year-old male reported in emergency with painful abdomen and diagnosed as having peritonitis. On laparotomy, we incidentally found giant and multiple jejunal diverticula along with ileal perforation. Nothing was done to the jejunal diverticula, as these were multiple and non-obstructive. In the follow-up of 16 months, the patient was doing well. Jejuno-ileal diverticulosis is a rare condition that continues to present formidable challenges in diagnosis and treatment.

## Introduction

Jejunal diverticulosis is a rare condition with variable clinical and anatomical presentations. The prevalence of small intestinal diverticula on autopsy ranges from 0.06% to 1.3% [**1**]. Although there is no consensus on the management of asymptomatic jejunal diverticular disease, some complications are potentially life threatening and require early surgical treatment [**2**]. Jejunal and jejuno-ileal localization is nearly three times less frequent than the duodenal, but of about four times likely to develop complications [**2**]. Asymptomatic cases require neither medical nor surgical treatment. Rarity of mild or chronic presentations explains the absence of clear consensus on therapeutic strategy and conservative management [**2**]. The majority of jejunal diverticulosis cases are discovered incidentally during radiological investigations. Surgical exploration is the treatment of choice for almost all acute complicated cases [**2**]. Most small bowel diverticula are thought to be acquired pulsion lesions. The clinical presentations of acquired jejunoileal diverticulosis are vague and diverse. As a result, identification of the disorder can be quite difficult. Presenting complaints such as intermittent abdominal pain, constipation and diarrhea, akin to those seen in irritable bowel syndrome, have been demonstrated in up to 90% of the patients and imaging tests have mostly atypical appearance without key diagnostic features and may not correlate with the clinical symptoms [**1**]. Thus, clinical recognition of the disease depends primarily on an awareness of the condition and the various ways in which it may present.

## Case report

A 63-year-old man reported in the emergency with generalized abdominal pain, with off and on episodes of vomiting. The patient also had a history of fever and constipation for two days. On physical examination, the temperature was of 36°C, heart rate of 110/min, blood pressure of 120/60 mmHg and the respiratory rate was of 18 breaths/min. Abdominal examination revealed generalized abdominal tenderness and signs of peritonitis. Bowel sounds were absent. Routine blood tests showed increased white blood cell (WBC 18.29 × 109/L), an impaired renal function (urea 14.2 mmol/L; creatinine 122 μmol/L). Abdominal X-ray revealed multiple dilated loops of small intestine and air fluid levels. 

**Fig. 1:**
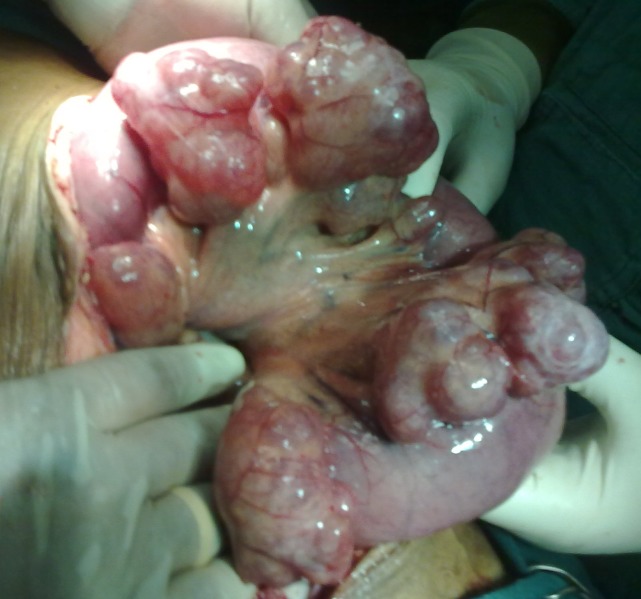
Multiple giant jejunal diverticula

**Fig. 2:**
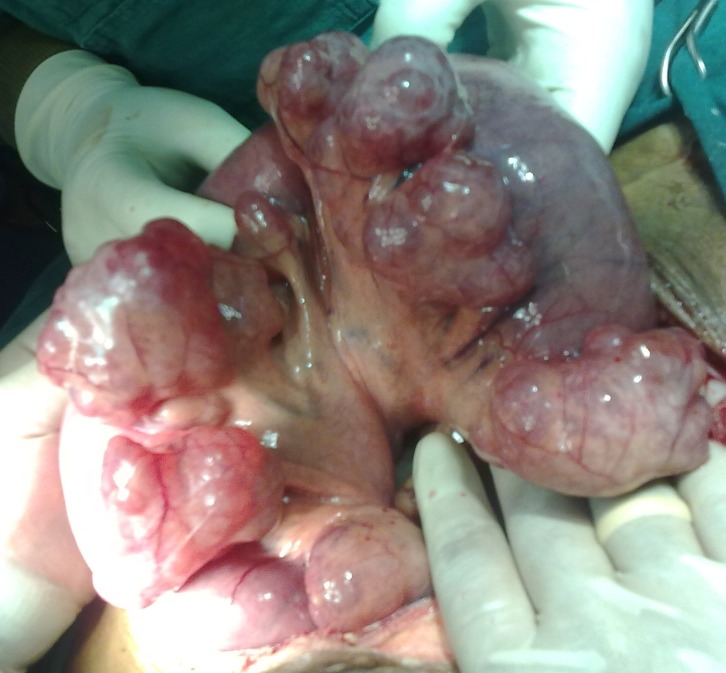
Multiple giant jejunal diverticula

The planned exploratory laparotomy identified a single perforation of the ileum associated with fecal contamination. To our surprise, multiple giant jejunal diverticula were seen (**Figures 1 and 2**)).

Perforation was primarily closed and the diverticulum was left behind, as they were not causing any trouble. A thorough abdominal washout was given. In the follow-up of 16 months, the patient was doing well and was asymptomatic. There are no complaints of obstruction. 

## Discussion 

Acquired jejunoileal diverticulosis was first described in 1794 by Sommering and later in 1807 by Sir Astley Cooper and is characterized by herniation of mucosa and submucosa through the muscular layer of the bowel wall (false diverticula) on the mesenteric border of the bowel [**3**]. They are more common in elderly males (58%). The commonly affected part of the small intestine by the diverticula is the proximal jejunum (75%), followed by the distal jejunum (20%) and the ileum (5%). Co-existent diverticula can be present in the colon (30-75%), duodenum (15-42%), esophagus (2%), stomach (2%) and urinary bladder (12%) of the patients [**4**]. Multiple diverticulosis of jejunum represents an uncommon pathology of the small bowel. The disease is usually silent and must be taken into consideration in cases of unexplained malabsorption, anemia, chronic abdominal pain or discomfort [**5**].

Rodrigez et al. [**6**] reviewed the literature and noted symptoms in 29% of the cases. Many symptoms may be misdiagnosed as dyspepsia or irritable small bowel. Edwards described a triad as 'flatulent dyspepsia' (epigastric pain, abdominal discomfort, flatulence one or two hours after meals). Anemia due to iron deficiency and megaloblastic anemia have often been reported and commonly attributed to malabsorpion, steatorreia, and vitamin deficit [**5**]. Malabsorpion could be justified by the non-synchronous peristaltic movement of the bowel, the dilation of the diverticula, the stasis of the intestinal content and the bacterial overgrowth [**7**].

In the etiopathogenesis of the jejunoileal diverticula, the current hypothesis focuses on abnormalities in the smooth muscle or myenteric plexus. Careful microscopic evaluation of jejunal specimens with diverticula has shown that these abnormalities are of three types: fibrosis and decreased numbers of normal muscle cells, consistent with progressive systemic sclerosis; fibrosis and degenerated smooth muscle cells, suggestive of a visceral myopathy; neuronal and axonal degeneration indicative of visceral neuropathy. Any of these abnormalities could lead to distorted smooth muscle contractions of the affected small bowel generating increased intraluminal pressure. The result is herniation of mucosa and submucosa through the weakest mesenteric site of the bowel wall with penetration induced by paired blood vessels from the mesentery [**1**]. 

Unfortunately, the only means of confirming a diverticulum source is by the cessation of the pain after surgical resection of the involved segment of small bowel. Asymptomatic jejunoileal diverticulosis does not require intestinal resection as seen in our case [**5**]. Jejunal diverticulosis is a challenging disorder from a diagnostic perspective, with no truly reliable diagnostic tests. 

Abdominal or chest radiographs may demonstrate evidence of perforation, such as free air under the diaphragm or free peritoneal air, evidence of intestinal obstruction, or evidence of ileus, including multiple air-fluid levels and bowel dilatation. CT may identify thickening or inflammation of the jejunum or localized abscess formation [**8**]. Today, multi-slice CT is very useful to make the diagnosis of jejunal diverticulosis and appears clearly superior to conventional enteroclysis for small intestine diseases [**9**].

The complications of jejunoileal diverticulosis are chronic abdominal pain, malabsorption, hemorrhage, diverticulitis, obstruction, and perforation, and occur in 10%-30% of the patients [**10**]. Jejunoileal diverticulitis is quite uncommon and has a mortality rate as high as 24% [**11**]. We report a case of uncomplicated jejunal diverticulits in conjunction with ileal perforation and review its presentation, clinical features, and management. Complications requiring surgical intervention occur in 8%-30% of the patients [**1**]. In our case, no surgical intervention was required for diverticulum as it was giant, multiple in nature and non-obstructive.

Jejunal diverticulosis in the elderly can lead to significant morbidity and mortality and so it should be suspected in those presenting with crampy abdominal pain and altered bowel habits. Once jejunal diverticulosis has been diagnosed, conservative medical management should be instituted to alleviate symptoms and reduce the risk of complications associated with diverticular disease.

## Conclusions

Jejunal diverticulosis in the elderly can lead to significant morbidity and mortality and so it should be suspected in those presenting with crampy abdominal pain and altered bowel habits. It should not be regarded as an insignificant finding and should be kept high in the differentials in older patients presenting with unexplained abdominal symptoms because it may lead to life-threatening complications and death. In the presence of complications, surgical resection with primary anastomosis is the preferred treatment option.
